# Rathke’s cleft cyst presentation mimicking craniopharyngioma: Case report

**DOI:** 10.1016/j.ijscr.2020.01.035

**Published:** 2020-02-06

**Authors:** Faisal A. Farrash, Maher Hassounah, Hala A. Helmi, Eyas Othman, Naif H. Alotaibi

**Affiliations:** a Neurosurgery Department King Faisal Specialist Hospital and Research Center, Riyadh, Saudi Arabia; b College of Medicine, Alfaisal University, Riyadh, Saudi Arabia; c Otolaryngology, Head & Neck Surgery King Faisal Specialist Hospital and Research Center, Riyadh, Saudi Arabia

**Keywords:** Rathke’s cleft cyst, Craniopharyngioma, Transphenoidal approach, Case report

## Abstract

**Introduction:**

Rathke’s cleft cysts (RCCs) are benign cystic lesions from the remnant cells of the craniopharyngeal duct within Rathke’s pouch. Anticipation of such condition will help planning the treatment course.

**Presentation of case:**

We present a case of a 46-year-old male, referred to our tertiary center with a history of seizures, confusion and agitation for the past two weeks. After thorough investigations, he was found to have hyponatremia with low serum cortisol and hypothyroidism. After being sedated, intubated and stabilized, Magnetic Resonance Imaging (MRI) brain showed relatively medium to large sellar lesion which was highly suspicious of a craniopharyngioma (CP). However, after performing an endoscopic transphenoidal surgical resection of the lesion, clinical diagnosis supported RCC.

**Discussion:**

The best treatment approach for CP is a complete surgical resection via a transphenoidal approach. Considering the fluid component of RCC, it may be treated with a more conservative surgery and does not necessitate a surgery as invasive as that of CP.

**Conclusion:**

Given the mixed solid and cystic nature of CPs, it is possible to confuse it with RCC and vice versa. The presented case highlights that Rathke’s cyst may be challenging in terms of pre-operative diagnosis, peri-surgical management and histopathological examination.

## Highlights

• Rathke’s cleft cysts are benign lesions from the remnants of the craniopharyngeal duct.
• Its diagnosis is often confused with craniopharyngioma.
• Our case of this cyst demonstrates the importance of proper diagnosis and management.

## 1 Introduction

Rathke’s cleft cysts (RCC) are benign cystic lesions from the remnants of the craniopharyngeal ducts within Rathke’s pouch. At autopsy, they are found in 13%–33% of patients [[1], [2], [3], [4]]. Most RCCs are found incidentally on imaging as they are most commonly asymptomatic [5,6]. However, if the cyst enlarges significantly, it may cause symptoms of compression including headache, visual changes, and pituitary dysfunction [3]. Diagnosis of RCC might be challenging as it might be confused with other lesions such as craniopharyngioma (CP) [3]. It is believed that overlapping pathological characteristics between RCC and CP might challenge the final diagnosis of both pathologies [6].

The case presented to our tertiary center highlights that Rathke’s cyst may be challenging in terms of pre-operative diagnosis, peri-surgical management and histopathological examination. The work has been reported in line with the SCARE guidelines [7].

## 2 Case presentation

A medically-free 46-year-old male was referred to our tertiary center with a history of a recent-onset generalized tonic clonic seizure two weeks prior to his presentation. He had five episodes of these generalized tonic clonic seizures, each lasting for approximately two minutes. One week later, he became confused as he could not recognize his family and became very agitated. His symptoms were not associated with any visual changes, fever, chills or neck stiffness. He had a positive history of on and off headache since he was 20 years old that did not require any medications. His past medical and surgical history, family history and drug history were all unremarkable.

Upon examination, he was found to be drowsy, agitated and confused but was able to obey commands. His Glasgow coma scale was 15/15. His vital signs were all within normal ranges. The rest of the neurological and the respiratory examinations were found to be unremarkable. He was put on leviteracetam 500 mg orally twice per day for his seizures as well as haloperidol 5 mg IM four times a day as needed for his agitation.

The patient was seen by endocrinology and was found to have a low adrenocorticotropic hormone (ACTH), a low PM cortisol, hyponatremia and a high thyroid stimulating hormone (TSH). Synacthen test was within normal range. He was put on hydrocortisone 20 mg q am and 10 mg q pm initially then decreased to 10 mg am and 5 mg pm as well as levothyroxine 75 mcg daily. Unfortunately, the patient started to develop extra-pyramidal symptoms presenting as abnormal movements in the lower limb and orofacial dyskinesia. It was believed to be caused by the haloperidol, which was therefore stopped. It was also believed that his agitation could be caused by leviteracetam, hence it was switch to sodium valproate 500 mg twice daily and clonazepam 1 mg once daily at bedtime.

**Fig. 1:**
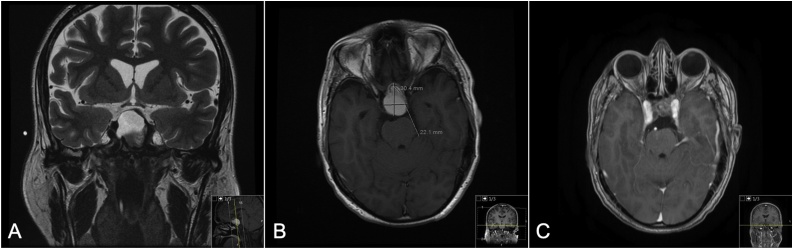
A: Pre-operative MRI T2 coronal section showing sellar/suprasellar lesion. B: Pre-operative MRI T1 with contrast axial section showing sellar/Suprasellar lesion. C: Nine months post-operative MRI T1 with contrast axial section with residual calcification.

After being sedated and stabilized, on MRI image, he was found to have a sellar/supra-sellar lesion measuring approximately 3.04 × 2.21 cm, predominantly cystic in nature with prominent calcification of the anterior wall. On T1 imaging, it was found to have a hyperintense signal. There was resultant expansion of the sella and scalloping of its walls as well as elevation of the optic pathway. The lesion was closely related to the anterior cerebral arteries, supra-clinoid internal carotid arteries and the basilar artery (Fig. 1A and B). Images were otherwise normal. EEG showed mild nonspecific generalized cerebral dysfunction with no epileptiform discharges identified. Based on MRI findings the patient was initially diagnosed with CP.

Pre-operatively, the patient required to have a stress dose of hydrocortisone 100 mg IV. Surgical transphenoidal approach was done for complete resection of the CP. The solid component of the lesion was sent for frozen section and the fluid was sent for cytology. The patient was shifted to the ICU in a stable condition. There were no surgical complications.

Cystic fluid cytology confirmed cytomorphological features consistent with cyst contents and negative for malignant cells. Histopathological examination of the supra-sellar lesion revealed microscopic focus of glandular tissue suggestive of salivary origin within a background of vascularized connective tissue. The supra-sellar tumor also revealed dense fibrous tissue with calcification, old hemorrhage, cholesterol clefts and hemosiderin laden macrophages.

Upon endocrinology, otolaryngology and neurosurgery clinic follow ups, the patient was found to be stable, seizure free, and without active complaints. He was still found to have panhypopituitarism and was maintained on hydrocortisone and levothyroxine. Post-operative EEG was normal. Post-operative MRI, done 9 months later, revealed residual calcified component at the anterior surgical margin, asymmetric to the right side in the supra-sellar region (with resultant elevation of the optic chiasm) and symmetric to the left side in the sellar region. There was no acute infarct, hydrocephalus or herniation syndrome (Fig. 1C).

## 3 Discussion

Rathke’s cleft cysts (RCC) are uncommon benign cystic lesions of epithelial remnants of the corresponding Rathke’s pouch with undetermined pathogenesis [8]. The incidence is variable with a peak in the third to fifth decade of life, in which our case falls [8]. Clinical manifestations vary from being completely asymptomatic to neurological and visual symptoms including headache, epilepsy, reduced vision, visual field defect and pituitary dysfunction [6,9,10]. Rao et al. observed in an ophthalmic study optic nerve atrophy in 8/11 cases, which might be considered in the examination of these cases and might necessitate early intervention [10].

CP is a benign tumor of central nervous system (CNS), which accounts for less than 1% of all CNS tumors. Although it’s considered a benign tumor, CP of sellar and suprasellar regions frequently causes invasion to neurovascular structures including pituitary gland and optic apparatus [11].

The patient reported in this case had typical history of headache followed by a recent onset of seizures and endocrinological disturbances. However, he did not have visual manifestations. His rapid deterioration of symptoms might be related to the enlarging cyst size that might reach up to 4 cm with accumulating fluid and gelatinous material [9,10]. Many reports have highlighted the difficulty in diagnosing RCC from other lesions in that area especially arachnoid cyst and CP preoperatively [12]. Patients with CP were found to have higher chance of presenting with hypopituitarism, neurological deficit and ophthalmological manifestations in 95%, 67% respectively [12]. Therefore, our patient was presumed to have CP preoperatively which may have affected our surgical plan and approach.

Radiological examination aids in the diagnosis of RCC, where a well-demarcated cystic lesion is usually identified with variable intensity by MRI depending on the cyst contents [8]. However, the presence of calcification and/or the finding of a solid component is more characteristic of CP, which was again a misleading feature in the diagnosis of our patient [12]. The histopathological examination will be the mainstay of the diagnosis with the typical finding of vascularized connective tissue, various epithelial types (more specifically the ciliated and mucous-secreting), and the peculiar fluid contents [9,12]. Therefore, clinical diagnosis of our case supports RCC.

In terms of the management, the recommended approach is trans-sphenoidal for drainage and biopsy of RCC. We have used the same surgical modality for our patient aiming at complete excisional biopsy [13]. It is believed that obtaining a sample by resection of the anterior portion of the cyst and fenestration might be the optimal surgical option during surgery [14,15]. CPs are treated with gross total resection or subtotal resection followed by radiotherapy [11]. The surgical objective in this reported case was total gross resection under the impression that the pathology is CP. The total gross resection was not necessary as the final diagnosis did not reveal CP. A less aggressive surgery to avoid potential post-operative risks including diabetes insipidus and CSF leak, might be enough in treating the patient and improve his presenting symptoms [16].

Surgical complications have been reported and include: meningitis (due to leakage of the cystic fluid into the subarachnoid space), CSF rhinorrhea, abscess formation and diabetes insipidus [9,17]. In this reported case, the patient did not develop any of these complications and he has been followed up with no evidence of recurrence, which has a higher chance after craniotomy rather than the trans-sphenoidal approach we have used [18]. In such recurrent cases, extensive excision of the wall and full removal of any contents are essential [18]. In Trifanescu et al. study, surgically treated patients with RCC were followed up for mean period of 4 years, the relapse rate was 48%, therefore a long follow up period by imaging is suggested [19].

In conclusion, it is of importance to be familiar with the diagnosis of RCC and CP, and for them to be kept in the differential diagnosis of sellar and supra-sellar lesions in order to allow proper surgical planning, to prevent neurological and/or ophthalmological morbidity and avoid possible complications. The final histopathological diagnosis is challenging, as in this reported case. Therefore, clinical diagnosis can be established in similar cases.

## Funding

The authors whose names are listed certify that they have no affiliations with or involvement in any organization or entity with any financial interest, or non-financial interest in the subject matter or materials discussed in this manuscript.

## Ethical approval

This case report has been approved by the HEC/IRB at King Faisal Specialist Hospital and Research Center according to the declaration of Helsinki.

## Consent

Informed consent was obtained by the patient for the publication of this case report and images.

## Author contribution

1. Faisal A. Farrrash: Substantial contributions to the conception or design of the work. Final approval of the version to be published.
2. Maher Hassounah: Substantial contributions to the conception or design of the work; or the acquisition.
3. Hala A. Helmi: Drafting the work or revising it critically for important intellectual content.
4. Eyas Othman : Substantial contributions to the conception or design of the work.
5. Naif H. Alotaibi: Substantial contributions to the conception or design of the work.

## Registration of research studies

NA.

## Guarantor

Dr. Faisal Farrash.

## Provenance and peer review

Not commissioned, externally peer-reviewed.

## Declaration of Competing Interest

The authors have no potential conflict of interest to declare.
